# Retrospective Molecular Survey on Bacterial and Protozoan Abortive Agents in Roe Deer (*Capreolus capreolus*) from Central Italy

**DOI:** 10.3390/ani12223202

**Published:** 2022-11-18

**Authors:** Valentina Virginia Ebani, Chiara Trebino, Lisa Guardone, Fabrizio Bertelloni, Giulia Cagnoli, Iolanda Altomonte, Paolo Vignola, Paolo Bongi, Francesca Mancianti

**Affiliations:** 1 Department of Veterinary Sciences, University of Pisa, Viale delle Piagge 2, 56124 Pisa, Italy; 2 Centre for Climate Change Impact, University of Pisa, Via del Borghetto 80, 56124 Pisa, Italy; 3 Struttura Semplice Section of Genoa-Portualità, Istituto Zooprofilattico Sperimentale del Piemonte, Liguria e Valle d’Aosta, 16129 Genoa, Italy; 4 Ambito Territoriale Caccia Massa (ATCMS), Largo Bonfigli 3/5, Aulla, 54011 Massa, Italy

**Keywords:** *Capreolus capreolus*, roe deer, abortive agents, *Coxiella burnetii*, *Toxoplasma gondii*

## Abstract

**Simple Summary:**

Reproductive disorders in ruminants may be related to bacterial and protozoan agents. Several studies have been carried out to determine the spreading of *Brucella* spp., *Chlamydia abortus*, *Coxiella burnetii, Salmonella enterica, Listeria monocytogenes, Neospora caninum,* and *Toxoplasma gondii* in domestic ruminants but not in deer. These pathogens are not only a problem for pregnant females because they may cause systemic infections in all animals. Furthermore, considering that all these bacteria as well as *T. gondii* are zoonotic agents, monitoring wild ruminants is pivotal to verify changes in the epidemiological scenario from a One Health perspective, too.

**Abstract:**

Bacterial and protozoan agents can determine abortion and other reproductive disorders in domestic ruminants, but data regarding their occurrence in wild ruminants are scanty worldwide, including in Italy. The aim of this retrospective study was to verify the occurrence of the main bacterial and protozoan abortive agents in 72 spleen samples previously collected from roe deer (*Capreolus capreolus*) living in mountain areas of Central Italy. All samples were collected and submitted to DNA extraction for other investigations. Molecular analyses were carried out on the DNA samples to detect *Brucella* spp., *Chlamydia abortus*, *Coxiella burnetii, Salmonella enterica, Listeria monocytogenes, Neospora caninum,* and *Toxoplasma gondii.* Three (4.16%) roe deer resulted PCR positive for *C. burnetii* and one (1.38%) for *T. gondii*. These findings suggest that roe deer living in the investigated areas do not act as important reservoirs of the searched agents. However, the tested animals lived in a closed area without contact with domestic animals that are usually involved in the epidemiology of the investigated pathogens. Monitoring of wild ruminants is pivotal to verify changes in the epidemiological scenario from a One Health perspective, too.

## 1. Introduction

Several infectious agents may cause abortion in both domestic and wild ruminants. Among abortive bacteria, *Brucella* spp., *Chlamydia abortus*, *Coxiella burnetii*, *Salmonella enterica,* and *Listeria monocytogenes* are the most relevant pathogens able to infect humans, too.

The brucella genus includes seven species of terrestrial origin: *B*. *abortus*, *B*. *melitensis*, *B*. *suis*, *B*. *canis*, *B*. *ovis*, *B*. *neotomae*, *B*. *microti*, *B*. *papionis,* and *B*. *vulpis* and two species of marine origin: *B*. *ceti* and *B*. *pinnipedialis*. Furthermore, the novel species *B*. *inopinata* has been isolated from a breast implant infection [1]. Brucella abortus and *B*. *melitensis* are the species most involved in reproductive disorders of livestock ruminants; however, these species can infect wild ruminants, too [2].

*Chlamydia abortus* is an obligate intracellular bacterium responsible for the disease called enzootic ovine abortion that causes relevant economic losses due to abortion and stillbirth in mainly small ruminants but also cattle. *Chlamydia abortus* can affect wild ruminants, too [3].

*Coxiella burnetii* is the etiologic agent of Q fever, a worldwide zoonosis transmitted by ticks of several species, even though the infection is usually acquired through inhalation of contaminated aerosol or ingestion of contaminated food, mainly raw milk and dairy products. The pathogen infects a wide range of animal species, including wild and domestic mammals, birds, and reptiles. However, the most common reservoirs are cattle, sheep, and goats, in which *C. burnetii* causes reproductive disorders [4].

*Salmonella* spp. are zoonotic enteropathogens that affect several domestic and wild animal species, including mammals, birds, and reptiles. Two species are known: *Salmonella enterica*, the most widespread, and *Salmonella bongori*. They are responsible for severe gastrointestinal forms in humans, economic losses in livestock due to intestinal disorders, septicemic forms, and abortion. Wildlife has been supposed to be involved in the epidemiology of salmonellosis [5], although data about reproductive disorders in these animals are not available.

*Listeria monocytogenes* is a ubiquitous Gram-positive bacterium isolated from several animal species, including mammals, birds, and fish. Ruminants are considered to be the most susceptible animals in which abortions frequently occur and the main reservoir for human infection. Listeriosis is one of the most severe food-borne diseases in humans [6].

The most frequent abortive parasites in ruminants are the apicomplexan protozoa *Neospora caninum* and *Toxoplasma gondii* [7]. *Neospora caninum* is responsible for abortions in bovine and ovine and neuromuscular paralysis in dogs [8,9,10]. This agent has a dixenous cycle, with dogs acting as the final hosts. They shed oocysts which sporulate in the environment producing sporozoites. These stages accomplish multiplications in the muscular and nervous tissues of intermediate hosts. The parasite can transmit vertically from mother to fetus by an endogenous cycle, bypassing the definitive host [7].

*Toxoplasma gondii* is strictly related to *N. caninum*, with cats acting as final hosts. Its life cycle is very similar, and herbivore intermediate hosts become infected mostly following the ingestion of oocysts shed by cats and sporulated in the environment. As for *N. caninum,* sporozoites are responsible for the cycle in both muscles and nervous tissue of intermediate hosts. Both parasites have a wide range of intermediate hosts, including mammals and avian species. Furthermore, *T. gondii* is zoonotic and responsible for miscarriage and ocular disease in immunocompetent people and severe encephalitis in immunocompromised human hosts [7,11].

The capability of these bacterial and protozoan agents to determine abortion and other reproductive disorders in domestic ruminants is well known, but data regarding their occurrence in wild ruminants are scanty worldwide. To the best of our knowledge, surveys showing the spreading of different abortive microorganisms in deer living in Italy are not available.

This is a retrospective study carried out on collected DNA from spleens of roe deer (*Capreolus capreolus*) living in mountain areas of Central Italy to investigate the occurrence of the main bacterial and protozoan agents responsible for reproductive disorders in domestic and wild ruminants. For this purpose, molecular analyses were carried out to detect *Brucella* spp., *C. abortus*, *C. burnetii, S. enterica, L. monocytogenes, N. caninum,* and *T. gondii.*

## 2. Materials and Methods

### 2.1. Animals

Molecular analyses were carried out on a total of 72 DNA samples previously extracted for other studies from spleen specimens collected from roe deer regularly hunted during the hunting seasons of June–September 2018 and 2019 in north-western Apennine, specifically in mountainous areas in Massa-Carrara and Lucca provinces (Central Italy). 

The animals had not undergone postmortem examinations because they were destined for meat consumption; only spleens were sampled. Spleen samples were collected by hunters; each sample was put into a sterile jar and brought in refrigeration conditions to the Department of Veterinary Sciences, University of Pisa within 24 h from the collection time. DNA was extracted from all spleens and stored at −20 °C.

The roe deer were 29 females and 43 males. Thirty-five (19 males and 16 females) were young (12–24 months), 24 adult males (>2 years), and 13 adult females (>2 years). The age classes were attributed as reported by Hoye [12]. 

### 2.2. Molecular Analyses

The DNA was previously extracted individually from about 10 mg of spleen with the DNeasy Tissue kit (Qiagen GmbH, Hilden, Germany) according to the manufacturer’s instructions and including extraction controls to monitor cross-contamination of samples. DNAs were stored at −20 °C until used in the PCR assays.

To detect DNA of *Brucella* genus members, a PCR assay with the primers B4 and B5 targeting a 223 bp fragment of the gene *bcsp31* was carried out [13].

Primers pmp-F and pmp-R821 were used to detect an 821 bp fragment of *C. abortus pmp90/91* gene [14].

PCR protocol for detection of *C. burnetii* employed the primers Trans-1 and Trans-2 that allow the amplification of a 687 bp fragment of the *IS1111* gene [14].

*Salmonella* spp. DNA was searched with primers invAF and invAR that allow the amplification of a 930 portion of the *invA* gene [15].

For *L. monocytogenes* DNA detection, the primers LIP1 and LIP2a amplifying a 274 bp fragment of the gene *prfA* were employed [16].

Primers Np6plus and Np21plus that amplify a 337 base pair fragment of the Nc5 region of *N. caninum* DNA were used as described by Muller et al. [17].

A nested PCR was carried out to amplify regions of the *B1* gene of *T. gondii* using outer and inner primers in the two steps, respectively, as described by Jones et al. [18]. Target genes, primers, and annealing temperatures used in each PCR protocol were summarized in Table 1.

For each PCR assay, negative and positive controls were included. Distilled water, instead of DNA, was used as negative control. Cultures of *L. monocytogenes*, *S. enterica* (serotype Typhimurium), and *Brucella ovis* were used to extract DNA to use as positive controls. Moreover, for *C. burnetii, C. abortus, N. caninum,* and *T. gondii*, DNA extracted from commercial IFAT slides (Fuller Laboratories Fullerton, Torrance, CA, USA) was included as positive controls.

## 3. Results and Discussion

The results obtained in the present molecular survey, with only three animals positive (4.16%; 95% CI: 0–8.77%) for *C. burnetii* and one (1.38%; 95% CI: 0–4.07%) for *T. gondii* (Table 2), suggest that roe deer living in the selected area do not act as important reservoirs of the investigated bacteria and protozoa. 

These findings could not reflect the real epidemiological scenario because testing multiple tissues could enhance case detection. On the other hand, even though abortion and other reproductive disorders are the main signs in pregnant animals infected by the investigated agents, these microorganisms cause systemic infections due to bacteraemic/parasitaemic phases, with spleen localization in all animals. Moreover, in other animal species, the recurrence of both parasite’s DNA in blood has been reported. This event can be ascribed to a possible resurgence of *T. gondii* tachyzoites from cysts in ewes [19] and endogenous cycles reported in cattle [20] and suspected in donkeys [21].

Furthermore, the obtained results seem not surprising because the tested roe deer lived in a closed area without contact with domestic animals that are usually involved in the epidemiology of the searched pathogens. However, some of the investigated microorganisms, mainly *Salmonella* spp. and *C. burnetii*, may be shed in the feces of wild birds [22,23,24], and consequently, they could contaminate the environments where ruminants are present.

Even though deer species are considered susceptible to the studied agents, data available in the literature about the spreading of these microorganisms in deer populations are very scanty worldwide and often concern the occurrence of antibodies in the analyzed animals; therefore, our results cannot be compared to other epidemiological scenarios.

The capability of these pathogens to cause abortion and other reproductive problems in domestic ruminants are well known, and it has been supposed that pathogenetic mechanisms are similar in wild ruminants, but data about the occurrence of abortions in these animals, that are not regularly controlled by humans, are not available.

All tested roe deer resulted negative for *Brucella* spp. In Italy, the prophylaxis plans have significantly reduced the circulation of brucellae, and Tuscany is considered an officially free territory according to community legislation [25]. The obtained results are related to the epidemiological situation, but they could also be influenced by the fact that wild ruminants are considered epidemiologic dead-end reservoirs [26]. In fact, some studies showed that wild ruminants do not play a relevant role in the maintenance of *B. abortus* and *B. melitensis* infections since limited cases of brucellosis have been reported in these animal populations [26,27]. Only weak evidence for a direct relationship between brucellosis and the size/density of the population of wild cervids has been reported in North America [28].

The negative results for *C. abortus* are not very surprising if compared with the scanty data about chlamydiosis in wild ruminants present in the literature. Only one case report describes abortion in a springbok (*Antidorcas marsupialis*) in Paris [29]. 

Available information is mainly based on serological investigations. In Italy, prevalences of 79% in fallow deer (*Dama dama*) [30] and 31% in alpine ibex (*Capra ibex*) [31] were found using the complement fixation test (CFT). Similarly, a CFT-based study in populations of wild ruminants, including fallow deer, red deer (*Cervus elaphus*), mouflon (*Ovis aries musimon*), and Iberian ibex (*Capra pyrenaica*), in southern Spain detected seroprevalence for *Chlamydiaceae* ranging from 24 to 37% and was higher in those areas where contact with domestic sheep and goats was likely [32]. With an ELISA test, Salinas et al. [33] found antibodies against *Chlamydiaceae* LPS (lipopolysaccharide) in 60% of red deer, 43.3% of fallow deer, and 28.3% of roe deer whereas when the same animals were tested with *C. abortus* POMP (polymorphic outer membrane protein) antigen, prevalences of 16.7%, 24.4%, and 20% were detected in the three deer species, respectively. A microimmunofluorescence assay found red deer positive for *C. psittaci* (9.6%) and *C. suis* (3.3%) but negative for *C. abortus* and *C. pecorum* [3].

All tested roe deer resulted negative for *Salmonella* spp., too. PCR assays carried out on spleen samples did not detect animals acting as fecal shedders, thus these findings cannot add complete information about the role of roe deer as sources of salmonellae for other animals. Previous studies showed that deer can be shedders of salmonellae with no apparent signs of illness. Roe deer as well as elk (*Cervus elaphus nelsoni*), sika deer (*Cervus nippon*), and white-tailed deer (*Odocoileus virginianus*) have been recognized as potential carrier animals of *Salmonella* serotypes [5,34,35].

All samples were negative for *L. monocytogenes.* This pathogen has been previously found in different specimens of healthy deer, such as in tonsils of 8/18 (44.4%) *C. elaphus hispanicus* in Spain [36] and the content of the rumen of red deer in Germany [37]. *Listeria monocytogenes* can cause disease in wild ruminants, too; it has been related to a large outbreak of meningo-encephalitis in fallow deer in Denmark [38] and the mortality in farmed fallow deer in Sweden [39].

*Coxiella burnetii* DNA resulted present in 3/72 (4.16%) roe deer. This finding shows that the pathogen is circulating in wildlife living in the selected area, though not largely. However, although the detected prevalence was lower than others found in red deer from Central Italy (10%) [40] and roe deer in the Netherlands (23%) [41], this value agrees with the 5.1% prevalence in roe deer in Northern Spain [42]. Circulation of *C. burnetii* in deer populations is related to the spreading of the bacterium among wildlife and ticks; in fact, *C. burnetii* is considered a tick-borne pathogen able to infect several wild species and transmitted by ticks mainly of the *Ixodes* genus. The ability of *C. burnetii* to cause reproductive disorders in deer has been studied mainly in farmed animals. It has been observed that *C. burnetii* DNA was still present in vaginal swabs of red deer five months after calving. This indicates that *C. burnetii* was shed in vaginal mucus for a long period after parturition or reproductive failure, constituting a source of environmental contamination and zoonotic Q fever [43].

As far as protozoans are concerned, only one roe deer (1.38%) resulted PCR positive for *T. gondii,* and all animals were negative for *N. caninum*.

Data from the distribution of *T. gondii* in roe deer populations from Europe would demonstrate high prevalences although all the studies were made by serological testing, as above reported for bacterial agents. The mean value in seroprevalence was determined as 29% [44]; however, these data range from 2% [45] to 52% [46]. To the best of our knowledge, there are no data concerning the risk of toxoplasmic abortion in roe deer; this parasite is reported to be associated with abortion in farmed red deer from New Zealand [47].

The results of the present study, obtained by PCR, are not easily comparable, considering that this method, together with the tissue samples employed, may negatively affect the prevalence estimation in comparison with serological determinations. The examined tissue specimens were not the most suitable to recover parasite DNA. An association between positivity to serological testing and PCR for *T. gondii* was in fact found when 50 g of heart muscle was employed [48]. Furthermore, detection methods for *T. gondii* infections should be further standardized to dispose of reliable epidemiological data [49]. However, the prevalence of the parasite in cervids is reported as very high, and any part of the carcasses should not be consumed raw, including the spleen [50], indicating that the tissue is considered a suitable site for *T. gondii.* So, considering the limits of the present study, the data gathered would indicate a low circulation of *T. gondii* among the selected roe deer population. The occurrence of toxoplasmic infection in wild ruminants is strongly affected by the ecosystem and management [45,51], and roe deer populations with a low density and living in arid areas are reported to be less prone to infection [52].

The lack of positivity for *N. caninum* is not surprising in such a scenario. Epidemiological data are available from a seromolecular study [46], which refers to a positivity of about 0.6% in Belgian roe deer together with a seropositivity of 2.7%. Molecular testing was carried out on brain samples, a more sensitive tissue to retrieve parasite DNA. Seroprevalences in Europe in roe deer range from very low (0.5%) [53] to higher (14%) [54]. Anyway, a lower seroprevalence of *N. caninum* antibodies with respect to *T. gondii* was reported from Spain [45], suggesting a scarce occurrence of sylvatic cycles in the study area. Furthermore, prevalence data from Western Europe suggest less intense parasitism by the studied protozoa with respect to those from Eastern Europe, indicating a higher circulation of the parasites in both domestic animals [55,56] and wildlife [57]. The animals were living, in fact, in a mountainous area where the other ungulate species present included wild boar (*Sus scrofa*) and red deer; mouflons and fallow deer were spotted on rare occasions and only in specific areas. Domestic ruminants are not present. In this environment, contact with the final hosts, such as cats and dogs, was unlikely, strongly reducing the occurrence of horizontal life cycles.

## 4. Conclusions

The present survey suggests that roe deer living in the investigated areas do not act as important reservoirs of the main bacteria and protozoa responsible for reproductive disorders in ruminants.

The tested wild ruminants lived in a closed area without contact with domestic animals, such as cattle, sheep, goats, dogs, and cats, which are the animal species most frequently involved in the epidemiology of the investigated bacteria. Furthermore, the low occurrence of sylvatic cycles of the protozoa in roe deer from the area of study would result in the low dispersal of populations along with a shortage of definitive hosts.

Monitoring wild ruminants for pathogens that can affect their health status and be transmitted to domestic animals is necessary to evaluate variations of the epidemiological scenario and possible risks of infections in a certain geographic area.

Considering that all investigated bacteria as well as *T. gondii* are zoonotic agents, these investigations are also useful from a One Health perspective. In fact, previous studies showed that wild ruminants can provide a bridge for the transmission of pathogens from wildlife into domestic livestock and humans [58]. Wild ruminants can contaminate the environments shared by persons for recreational or work activities as well as can directly be sources of infections for hunters during the carcasses’ manipulation. 

## Figures and Tables

**Table 1:** Target genes, primers, and annealing temperature for the PCR assays carried out to detect DNA of each pathogen.

Pathogen	Target Gene	Primers Sequences (5′-3′)	Amplicons (bp)	Annealing Temperature	Ref.
*Brucella* spp.	*bcsp31*	B4: TGGCTCGGTTGCCAATATCAA B5: CGCGCTTGCCTTTCAAGGTCTG	223	60 °C	[13]
*Chlamydia abortus*	*pmp90/91*	pmp-F: CTCACCATTGTCTCAGGTGGA pmp-R821: ACCGTAATGGGTAGGAGGGGT	821	63 °C	[14]
*Coxiella burnetii*	*IS1111*	Trans-1: TATGTATCCACCGTAGCCAGT Trans-2: CCCAACAACACCTCCTTATTC	687	64 °C	[14]
*Listeria monocytogenes*	*prfA*	LIP1: GATACAGAAACATCGGTTGGC LIP2a: GTGTAATCTTGATGCCATCAGG	274	53 °C	[16]
*Neospora caninum*	*Nc5*	NP21: CTCGCCAGTCAACCTACGTCTTCT NP6: CCCAGTGCGTCCAATCCTGTAAC	337	63°C	[17]
*Salmonella* spp.	*invA*	invAF: GTTGTACCGTGGCATGTCTG invAR: GCCATGGTATGGATTTGTCC	930	50 °C	[15]
*Toxoplasma gondii*	*B1*	B1outF: GGAACTGCATCCGTTCATGAG B1outR: TCTTTAAAGCGTTCGTGGTC	193	57°C	[18]
		B1inF: TGCATAGGTTGCAGTCACTG B1inR: GGCGACCAATCTGCGAATACACC	96	62.5 °C	

**Table 2:** Data about the roe deer resulted PCR positive.

Roe Deer			
Identification Number	Sex	Age	PCR Results
RD4	Male	Adult	*Coxiella burnetii* positive
RD5	Female	Adult	*Toxoplasma gondii* positive
RD16	Male	Young	*Coxiella burnetii* positive
RD22	Female	Adult	*Coxiella burnetii* positive

## Data Availability

The data presented in this study are available within the article.
